# Brief high fat high sugar diet results in altered energy and fat metabolism during pregnancy in mice

**DOI:** 10.1038/s41598-020-77529-6

**Published:** 2020-11-30

**Authors:** Kathleen A. Pennington, Yuanlin Dong, Simone Hernandez Ruano, Nicola van der Walt, Haleh Sangi-Haghpeykar, Chandrasekhar Yallampalli

**Affiliations:** Basic Sciences Perinatology Research Laboratories, Department of Obstetrics and Gynecology, Baylor College of Medicine, 1102 Bates Street, Room #1850.36, Houston, TX 77030 USA

**Keywords:** Physiology, Diseases

## Abstract

During pregnancy several maternal adaptations occur in order to support the growing fetus which are further exacerbated by gestational diabetes mellitus (GDM). Previously we developed a mouse model of GDM, however we did not evaluate alterations to energy and fat metabolism. We have also shown that alterations in lipid metabolism are mediated by adrenomedullin (ADM) in normal and GDM pregnancies. Our objectives were: (1) evaluate energy and fat homeostasis in our GDM mouse model and (2) determine if ADM may play a role in these changes. Female mice were placed on either control (P-CD) or high fat, high sucrose diet (P-HFHS) 1 week prior to and throughout pregnancy. Mice were placed into comprehensive lab animal monitoring system (CLAMS) chambers throughout pregnancy. Visceral adipose tissue (VAT) was collected at d17.5 of pregnancy for analysis. Energy Expenditure was significantly increased (p < 0.05) in P-HFHS dams compared to all other groups. VAT ex-vivo lipolysis was increased (p < 0.05) in P-HFHS compared to P-CD dams. VAT gene expression of ADM receptors *Crlr*, *Ramp2*, and *Ramp3* was increased (p < 0.05) in P-HFHS dams. ADM dose dependently increased ex vivo lipolysis. This data further validates our animal model of GDM and is usefulness in investigating the pathophysiology of GDM.

## Introduction

Gestational diabetes mellitus (GDM) is one of the most commonly observed obstetrical complications, affecting 7–18% of all pregnancies^1,2^. GDM-related maternal adipose tissue dysfunction, including increased lipolysis, elevated maternal triglyceride levels, decreased HDL cholesterol levels and abnormal production of adipokines^3–6^ is associated with a significant risk of having large for gestational age (LGA) babies, and is a stronger predictor of fetal overgrowth than glucose levels^7^. Controlling glucose in GDM patients does not always results in normal size fetus^8,9^. Thus, impaired maternal lipid metabolism has profound and detrimental effects on fetal adiposity and overgrowth highlighting the need to further understand adipose tissue lipid metabolism during normal and GDM pregnancies.

Due to the transient nature of GDM, and the challenges to safely sample during pregnancy, studying the patho-physiology of the disease in women is difficult. Therefore, good animal models are necessary^13^. We recently developed a novel mouse model of GDM, where dams exposed to a brief high fat, high sugar (HFHS) diet 1 week before and throughout pregnancy develop multiple GDM-like symptoms including normal glucose tolerance prior to mating, glucose intolerance during pregnancy, and return to normal glucose tolerance post-partum^14^. Dams exposed to brief HFHS also have increased leptin and liver triglycerides compared to control dams^14^.

Adrenomedullin (Adm), a multi-functional protein, has been previously shown to have a role in mediating adipose tissue dysfunction^15–17^. Adm is a member of the calcitonin gene-related peptide (Cgrp) superfamily^18^, and its signal is mediated by calcitonin receptor-like receptor (Crlr)^19^. Crlr interacts with receptor activity-modifying protein (Ramp) 2 and Ramp3 to confer high affinity for Adm^19^. Adm and its receptor components^20,21^ are expressed in adipose tissue and may play a pathophysiological role in obesity^22^. Adm levels in adipose tissue are elevated in obese and high-fat diet fed mice models^20^ and in humans with obesity^23^ and T2DM^24^. We have shown that treatment of diet induced diabetic non pregnant female mice with Adm antagonist, Adm_22–52,_ reduces visceral fat lipolysis^17^. We have also shown that ADM plays a role in GDM related adipose tissue dysfunction using human explant tissue culture^15,16^. ADM levels are higher in plasma of pregnant compared to non-pregnant women^25,26^ as well as in the serum and amniotic fluid of GDM women^16,27^ Together this data supports a role for ADM in lipid metabolism during GDM.

Gaps remain in our understanding of the mechanisms regulating the pathophysiology of GDM, specifically with regards to adipose tissue dysfunction and homeostasis along with the role Adm may play. Here our objectives were two-fold: (1) evaluate energy and fat homeostasis in our GDM mouse model and (2) assess the possible role of Adm in mediating these changes. We hypothesized that GDM dams would have altered energy and fat metabolism and that Adm signaling would be involved in these changes. To test this we analyzed energy expenditure, visceral fat gene expression, ex vivo lipolysis as well as the Adm signaling system in our mouse model of GDM.

## Methods

### Animals and experimental design

All animal procedures were approved by the Baylor College of Medicine institutional animal care and use committee and performed in accordance with NIH Guide for the Care and Use of Laboratory Animals.

**Figure 1:**
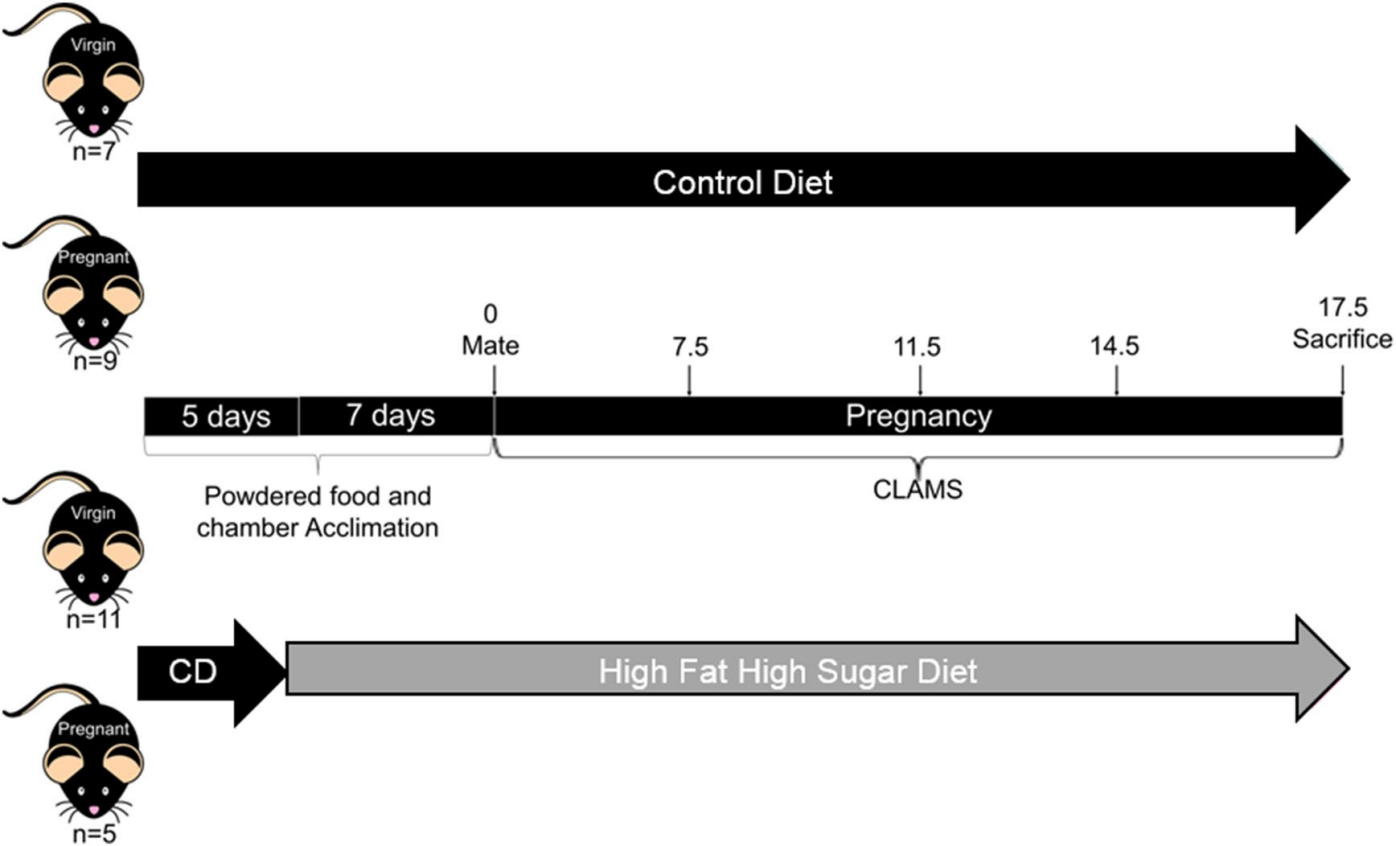
Experimental design. Mice were acclimated to powdered food and CLAMS chambers beginning 12 days prior to mating. Seven days prior to mating mice were randomly assigned to either CD or HFHS. On day − 1 mice were mated with proven breeder males for one night, mice where a copulatory plug was detected were considered pregnant and those where no copulatory plug was detected were designated non-pregnant. The day of copulatory plug detection (day 0) mice were placed in CLAMS cages for EE, food intake, and activity level measurements until day 17.5 pregnancy. At days 0, 6. 5, 10.5, 13.5 and 17.5 mice were briefly taken out of the cages, weighed, cages were cleaned, and then re-sealed. On day 17.5 mice were sacrificed and VAT was collected for further experiments.

Seven week old C57BL/6J female mice were placed on either a 10% kcal/fat, 0% kcal/sucrose control diet (Research Diets Inc., New Brunswick, NJ, cat#D12451; CD, n = 16) or a 45% kcal/fat, 17% kcal/sucrose diet (Research Diets Inc, cat #D12450K; HFHS, n = 16) 1 week prior to and throughout pregnancy to induced GDM like symptoms as previously described^40^. All females were placed with a proven breeder male for one night and then examined for copulatory plugs in the morning. Plug positive females were considered pregnant (P) and the morning of plug positive was designated as day 0.5 of pregnancy. Females who were plug negative were considered non pregnant (NP) and remained on their respective diets through-out the experiment to determine pregnancy vs diet specific effects. In total we had n = 7 NP-CD, 9 P-CD, 11 NP-HFHS, and 5 P-HFHS. Mice were placed into comprehensive lab animal monitoring system (CLAMS) chambers from day 0.5 to 17.5 of pregnancy to measure food consumption, activity, and energy expenditure (EE). Body composition was analyzed on day 0, 7.5, 11.5, 14.5 and 17.5. On day 17.5 animals were sacrificed using CO^2^ inhalation. NP animals were day matched to P animals and evaluated at the equivalent time points. Visceral adipose tissue (VAT) from the periovarian fat pad was collected for gene expression analysis and ex-vivo culture with and without ADM. See Fig. 1 for experimental timeline.

### Comprehensive Lab Animal Monitoring System (CLAMS)

Mice were placed into CLAMS (Columbus Instruments) in the Mouse Metabolic Research Unit at the USDA/ARS Children’s Nutrition Research Center from gestational day (d) 0.5 to 17.5, during which food intake, activity, and energy expenditure (EE) were measured as described previously^41–43^. Mice were first acclimated to the CLAMS for 3 days cages prior to breeding, followed by one night of mating in home cages. Mice were checked for copulatory plug and both mated and unmated mice were placed in CLAMS cages from d 0.5 to 17.5. Ad libitum food and water were provided throughout. Room temperature was maintained at 23.5 °C with a 12 h ligh/dark cycle. Data was analyzed using CLAMS data eXamination Tool (CLAX) (version 2.1.0; Columbus Instruments). On d 0.5, d 6.5, d 10.5, d 13.5 and d 17.5, cages were changed and body composition was measured with a Quantitative Magnetic Resonance (QMR) analyzer (EchoMedical, Houston Texas).

### Quantitative real-time PCR

Total RNA was isolated from VAT using TRIzol (Life Technologies, Grand Island, NY, USA) as previously described^44^. Reverse transcription was performed using the RT^2^ First Strand Kit (Qiagen) according to manufacturer’s instructions. PPAR target RT^2^ Profiler PCR array (Qiagen) was performed to analyze 84 unique PPAR gene targets according to manufactures instructions (n = 4/group).

For Adm signaling components quantitative Real-time-PCR was performed on VAT RNA (n = 5–6/group) using Taq universal SYBR Green Supermix (Bio-Rad). PCR primers used for amplification were purchased from Integrated DNA Technologies (IDT): *Adm* (Mm.PT.58.11111908), *Crlr* (Mm.PT.58.10636953), *Ramp2* (Mm.PT.58.30553776), *Ramp3* (Mm.PT.58.8586280) as previously described^17^.

### Adipose tissue explant culture

VAT obtained from mice was finely diced and transferred to wells of 24-well plates containing 1 ml of DMEM with 4.5 g/L d-glucose (Gibco, Life technology, Gaithersburg, MD), and cultured in a humidified atmosphere of 21% O_2_ and 5% CO_2_ at 37 C for 1 h as previously described^17,45^. After refreshing the medium, the tissues were incubated with or without increasing doses of Adm for 24 h and culture medium was collected for glycerol analysis. The glycerol level in culture medium was assessed using Free Glycerol Reagent (Sigma Aldrich, St. Louis, MO, USA) according to manufacture instructions. The absorbance at A540 were read and recorded by a Spectrophotometer CLARIO STAR (BMG Labtech, Inc., Cary, NC, USA) as previously described^17^.

### Statistical analysis

EE, activity, and food intake were analyzed using a repeated measures function in SAS (SAS Institute, Cary, NC, USA) with pregnancy status and diet as factors. Body weights, lean mass, and fat mass were analyzed with a two-way ANOVA with diet and time as factors with a Bonferroni post hoc analysis for comparison between groups using GraphPad Prism Software. Qiagen RT^2^ Profiler PCR array statistics were performed on calculated dCT using a two-way ANOVA with diet and time as factors with a Bonferroni post hoc analysis for comparison between groups using GraphPad Prism. Lipolysis and Adm signaling mRNA gene expression was analyzed by student t-test using GraphPad Prism. ADM gene expression statistical analysis was performed on calculated dCT. A one-way ANOVA was used to analyze ADM dose response effect on lipolysis using GraphPad Prism. Statistical significance was defined as p < 0.05 and data are presented as mean ± SEM.

## Results

### GDM results in increased energy expenditure

**Figure 2:**
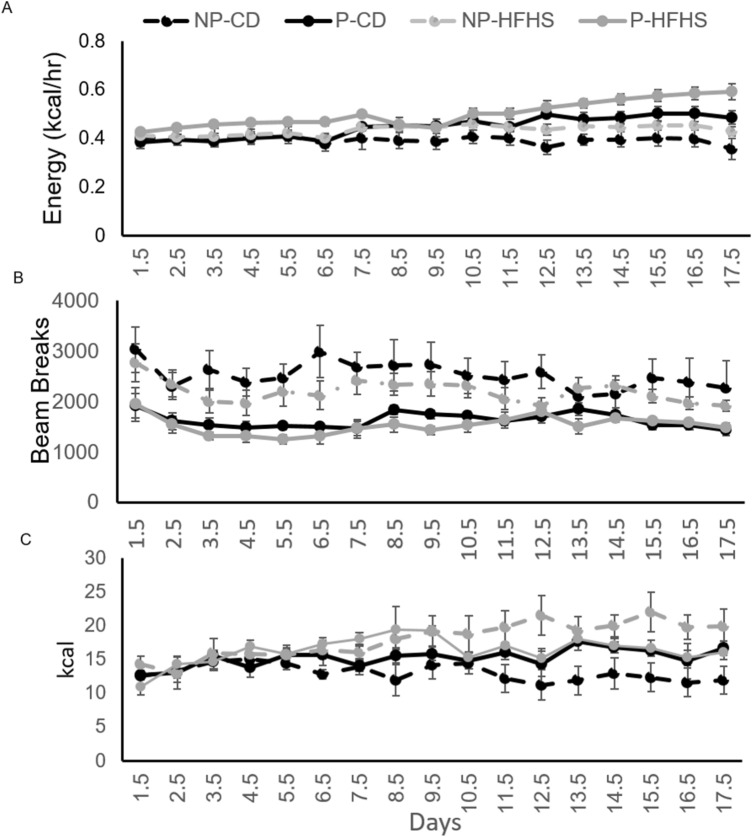
Brief HFHS diet exposure results increased energy exposure (EE). EE (**A**), locomotor activity (**B**), and food intake (**C**) were measured from day 1.5 through day 17.5 of pregnancy in NP-CD, P-CD, NP-HFHS, and P-HFHS dams. For EE (**A**) there was a significant interaction of diet*pregnancy*day (p = 0.0015). Activity (**B**) was significantly decreased in P vs NP females (p < 0.0001) and in HFHS vs CD fed females (p < 0.01). For food intake there was an interaction of diet*pregnancy (p < 0.0007). n = 7(NP-CD), 9 (P-CD), 11 (NP-HFHS) and 5 (P-HFHS); error bars ± SEM.

CLAMS was used to measure energy expenditure (EE), activity and food intake throughout pregnancy. There was a significant interaction of diet*pregnancy*day (p = 0.0015) in EE (Fig. 2A). Pregnant females had increased (p < 0.001) EE compared to non-pregnant females, regardless of diet. EE was increased in P-HFHS dams compared to all other groups (p < 0.001).

Overall activity was decreased in pregnant females compared to non-pregnant females (p < 0.0001) and in HFHS fed females compared to CD females (p < 0.01, Fig. 2B). Activity was decreased in NP-HFHS females compared to NP-CD females (p = 0.005), but not different between P-HFHS and P-CD females (Fig. 2B).

There was a strong interaction of diet*pregnancy on food intake (p < 0.0007) as measured by kcal (Fig. 2C). Food intake was decreased in P-HFHS dams compared to P-CD dams (p = 0.0005), but food intake was not different between NP-CD and NP-HFHS females. Food intake was significantly decreased in NP-CD females compared to P-CD dams (Fig. 2C).

**Figure 3:**
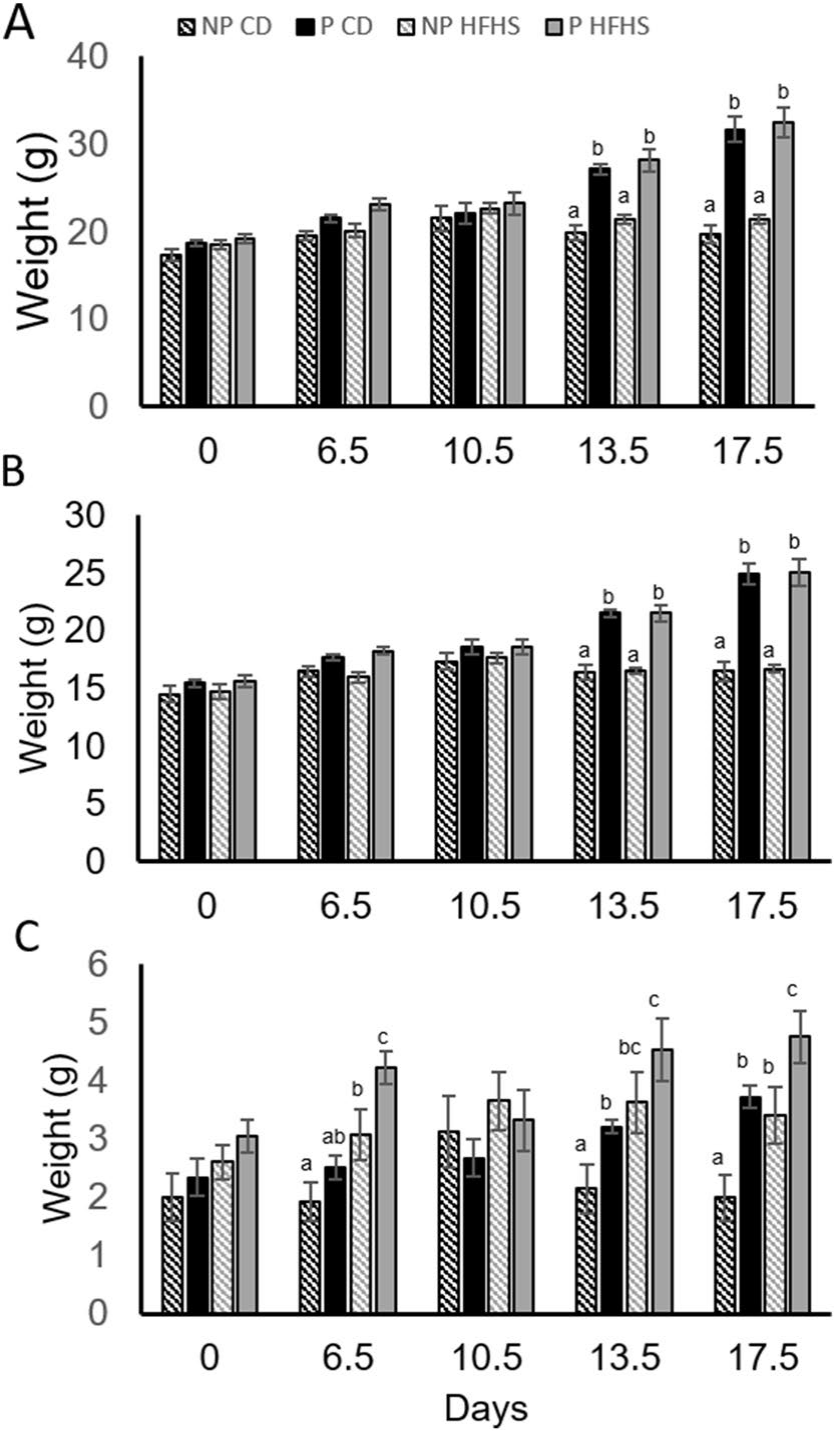
Body weight was not altered by HFHS diet. Body weight (**A**) and lean mass (**B**) were increased (p < 0.05) at day 13.5 and 17.5 of pregnancy in P vs NP females regardless of diet. Fat mass (**C**) was increased in P-HFHS diet compared to all other groups at day 6.5, 13.5 and 17.5 of pregnancy. n = 7(NP-CD), 9 (P-CD), 11 (NP-HFHS) and 5 (P-HFHS); difference subscripts represent differences among groups (p < 0.05); error bars ± SEM.

Notably body weight (Fig. 3A) and lean mass (Fig. 3B) were increased (p < 0.05) in pregnant vs non-pregnant females regardless of diet on days 13.5 and 17.5 of pregnancy (Fig. 3A) however there was no difference in body weight between P-CD and P-HFHS dams at any time point. Fat mass (Fig. 3C) was significantly increased (p < 0.05) in P-HFHS dams compared to all other groups on days 6.5, 13.5 and 17.5 of pregnancy. On day 6.5 NP-HFHS females had increased (p < 0.05) fat mass compared to NP-CD and P-CD females, while on days 13.5 and 6.5 NP-HFHS and P-CD had increased (p < 0.05) fat mass compared to NP-CD (Fig. 3C).

### Pregnancy alters adipose tissue gene expression and these changes are ablated in GDM dams

**Figure 4:**
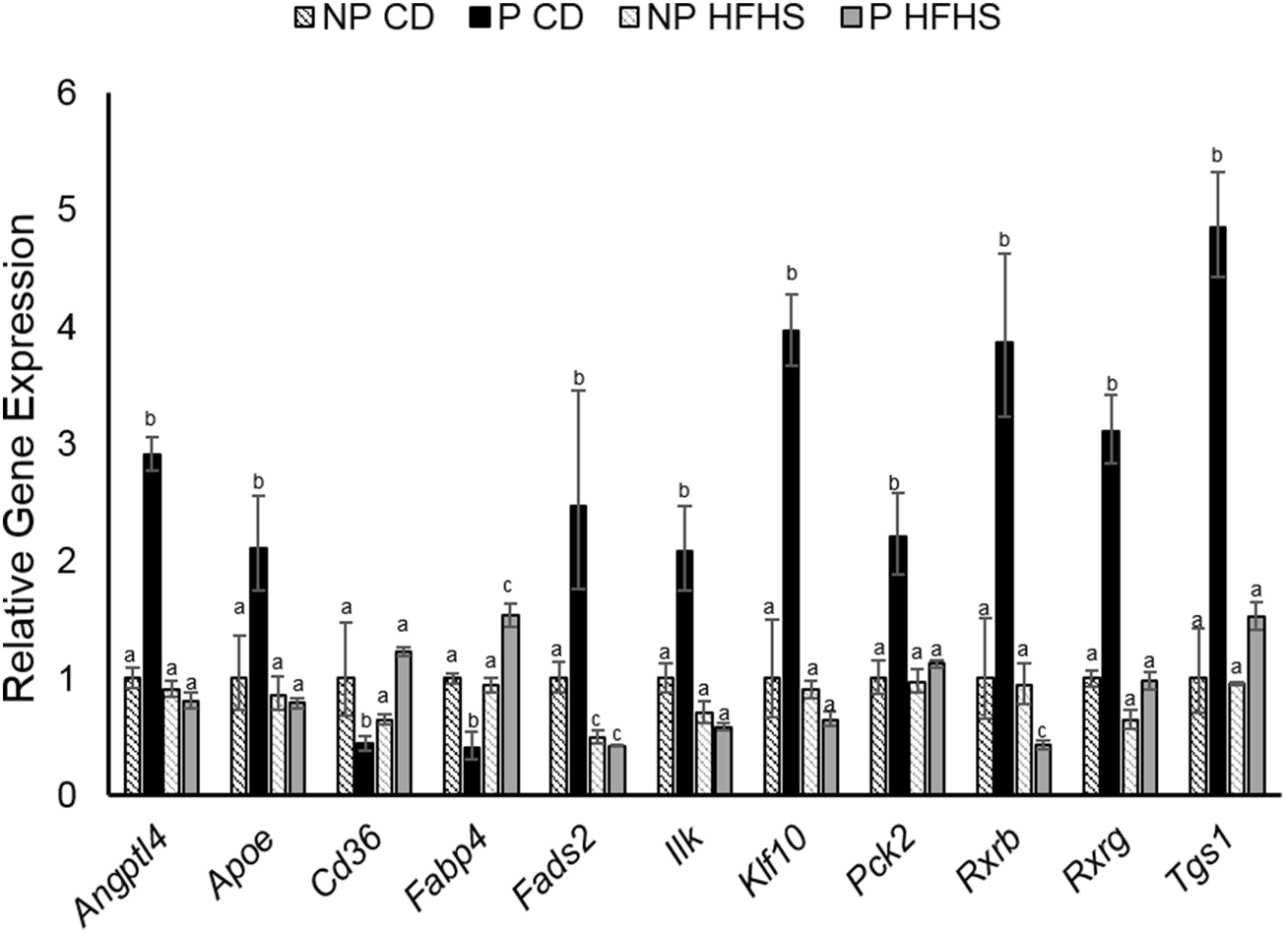
Brief HFHS diet alters pregnancy induced VAT gene expression changes at day 17.5 of pregnancy. Qiagen Ppar RT2 PCR profiler array were performed on n = 4 VAT samples from each group. Data presented as fold effect. Difference subscripts represent differences among groups (p < 0.05).

VAT was analyzed for differences in gene expression for peroxisome proliferator-activated receptor (Ppar) targets using Qiagen RT^2^ profiler arrays. Ppar signaling is a major player in fatty acid metabolism, adipogensis, lipid transport and insulin signaling^28–30^. Of the 84 genes analyzed we found that angiopoietin like 4 (*Angptl4)*, apolipoprotein E (*Apoe*), fatty acid desaturase 2 (Fads2), Integrin linked kinase (Ilk), krupple like factor 10 (Klf10), phosphoenolpyruvate carboxykinase 2 (Pck2), retinoid X receptor beta (Rxrb) and gamma (Rxrg), and trimethylguanosine synthase 1 (Tgs1) were significantly increased while, *Cd36* and fatty acid binding protein 4 (*Fabp4)* were significantly downregulated in P-CD dams compared to all other groups indicating that normal pregnancy alters Ppar signaling, while GDM like conditions suppresses these changes (Fig. 4).

### Lipolysis is increased in GDM dams which may be mediated by ADM

**Figure 5:**
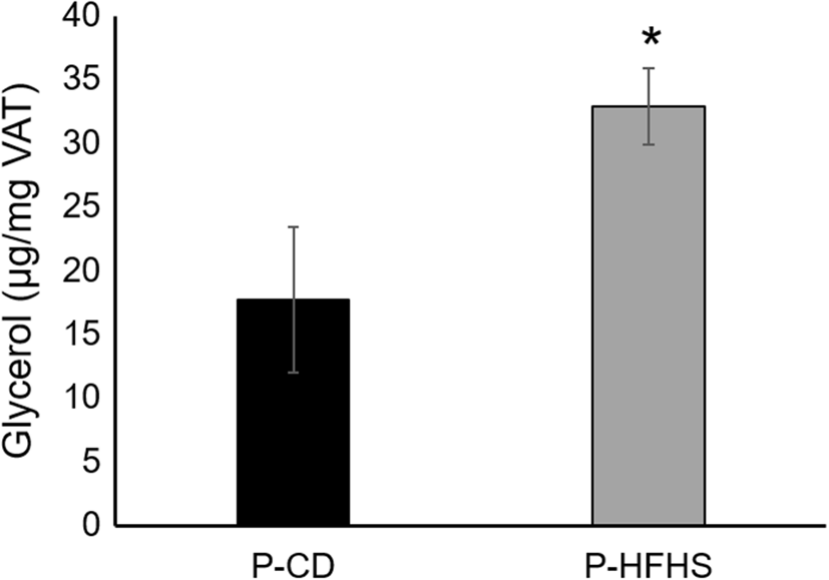
Lipolysis is increased in GDM Dams. Glycerol release was measured in ex vivo culture media of VAT from control and GDM dams (n = 6 P-CD and 5 P-HFHS). *p < 0.01, Bars are mean ± SEM.

Based on the gene expression and energy expenditure findings we decided to continue our experiments only in pregnant samples. VAT lipolysis was measured as glycerol release into the culture media after 24hrs ex vivo incubation of visceral fat from P-CD and P-HFHS dams (Fig. 5). Lipolysis was significantly increased (p = 0.0005) in P-HFHS dams compared to P-CD dams.

**Figure 6:**
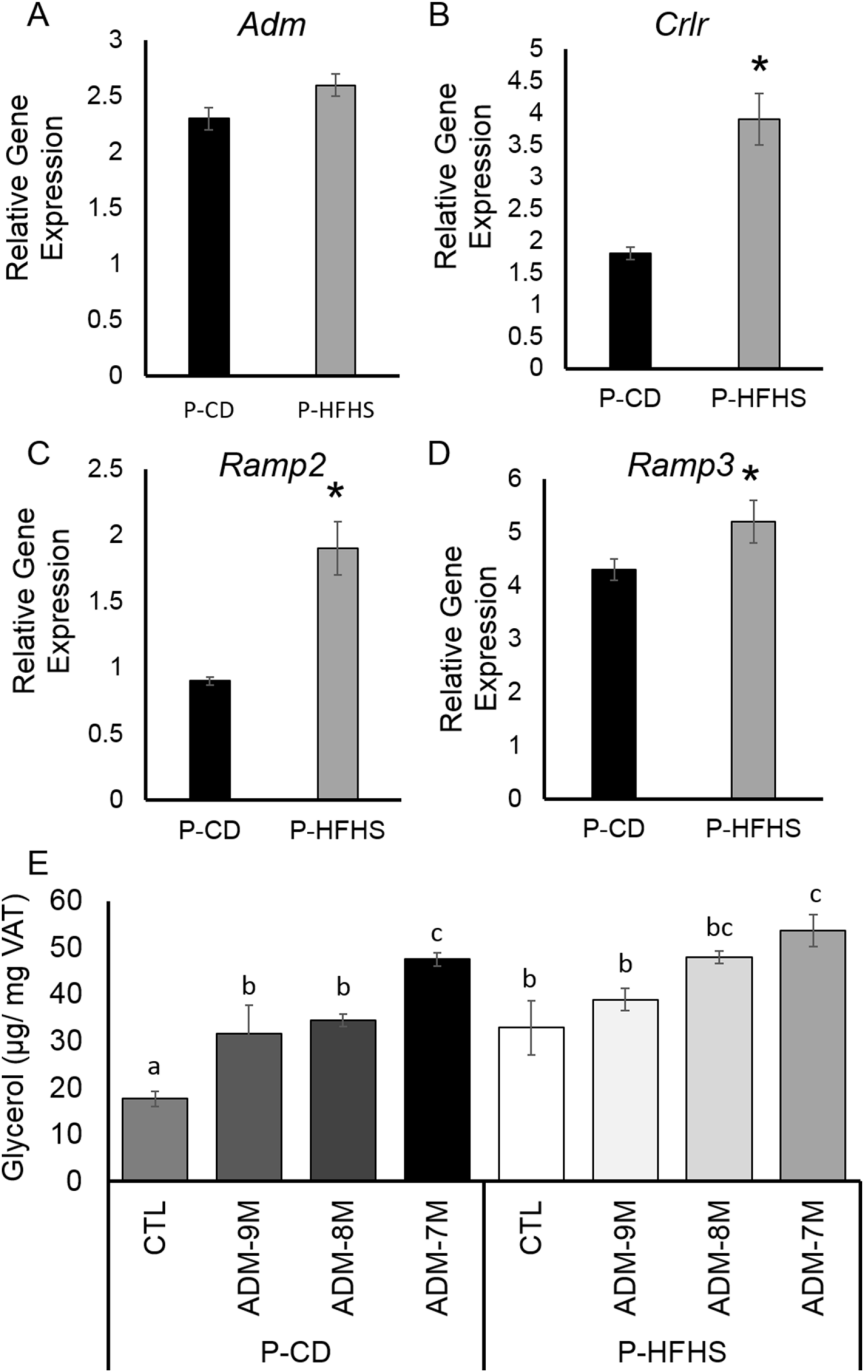
Analysis of Adm signaling in P-CD and P-HFHS Dams. Adm signaling is increased in VAT from GDM dams at day 17.5 of pregnancy. VAT mRNA expression of (**A**) *Adm*, (**B**) *Crlr*, (**C**) *Ramp2*, and (**D**) *Ramp3*. (E) Glycerol release was measured in ex vivo culture media of visceral fat from P-CD and P-HFHS dams w/o Adm after 24hrs as a measure lipolysis. Adm increased lipolysis in a dose dependent manner in both P-CD and P-GDM dams. *p < 0.05, different subscripts represent differences among groups (p < 0.01); n = 6 P-CD and 5 P-HFHS, error bars ± SEM.

As we have previously demonstrated ADM plays a role in increased lipolysis associated with GDM in human VAT^15,16^, we sought to determine if Adm may play a role in increased lipolysis in our mouse model. First, we assessed if ADM signaling system is increased in VAT. mRNA expressions for *Crlr* (Fig. 6B), *Ramp2* (Fig. 6C), and *Ramp3* (Fig. 6D) were increased in P-HFHS dams compared to P-CD dams, while ADM expression was not different between groups (Fig. 6A). We then assessed if Adm can induce lipolysis in VAT from P-CD and P-HFHS dams. Glycerol release was measured in media of VAT from P-CD and P-HFHS dams incubated ex vivo with increasing doses of Adm for 24 h (Fig. 6E). Adm increased (p < 0.05) lipolysis in a dose dependent manner in both P-CD and P-HFHS dams. Overall, P-HFHS dams had increased (p < 0.01) lipolysis compared to P-CD dams. Taken together this data indicates that Adm may play a role in the mechanisms regulating increased lipolysis observed in GDM dams.

## Discussion

Previously we showed that a brief HFHS diet 1 week before and during pregnancy resulted in glucose intolerance, decreased beta cell numbers and serum insulin levels, and increased leptin and triglyceride levels. Here we demonstrate that brief exposure to HFHS diet 1 week before and during pregnancy results in increased maternal EE possibly due to alterations in fat metabolism as indicated by alterations to VAT gene expression as well as increased lipolysis. These findings are consistent with observations in women with GDM^31,32^ further validating this as a valuable and novel animal model of GDM. Furthermore we demonstrate that ADM, as shown in human studies^15,16^, may play a role in altered lipid homeostasis during pregnancy as well as in GDM as indicated by increased receptor mRNA expression and an ability to dose dependently increase lipolysis.

During pregnancy energy expenditure increases and energy intake also increases to meet this increased demand^33^. To our knowledge this is the first study that evaluated energy expenditure throughout pregnancy in mice using CLAMS cages. Our data shows that in mice, both energy expenditure and energy intake are increased during pregnancy, indicating that mice are a useful model in evaluating energy balance during pregnancy. Furthermore, our data indicate that while P-HFHS dams had increased energy expenditure compared to all other groups, their energy intake was not different from P-CD dams. This data would indicate that P-HFHS dams are in a negative energy balance compared to P-CD dams, with increased EE but not energy intake.

There are several possible reasons for altered energy homeostasis in P-HFHS dams. Mice with glucose intolerance are often found to have increased energy expenditure^34^. However altered glucose metabolism alone does not completely explain the observed differences in P-HFHS dams. Another major contributor to energy homeostasis is lipid metabolism^35^. Lipid metabolism is known to be increased in pregnancy and even further increased in GDM^5^. We therefore sought to examine possible changes in lipid metabolism to determine if, altered lipid metabolism may also contribute to increased EE in P-HFHS dams.

Analysis of VAT gene expression showed that several genes involved in lipid metabolism were altered in normal pregnancy (P-CD dams) and that these alterations were diminished in GDM (P-HFHS dams). These findings indicated that normal pregnancy increased the gene expression of overall lipid metabolism regulators, *Angptl4, Ilk, Klf10, Pck2, Rxrb, Rxrg*, and *Tgs1*, while these factors were not increased in GDM dams. Interestingly a recent study linked Angptl4 to increasing triglyceride levels in pregnancy, and also found that diet induced obese pregnant mice had impaired Angptl4 expression^36^. This data corroborates our observation that *Angptl4* was increased in P-CD dams but not P-HFHS dams and potentially plays a role in altered lipid metabolism observed in P-HFHS dams.

Another key group of factors that were altered in P-CD were genes that play a specific role in fatty acid metabolism including, *Apoe*, *Cd36*, *Fabp4*, and *Fads2*, however these genes were not altered in P-HFHS dams. These same findings were not observed between NP-CD and NP-HFHS females indicating that these changes were specific to pregnancy and not diet. Decreases in Cd36, which is involved in fatty acid uptake have previously been reported in omental fat tissue from healthy, non-obese and normal glucose tolerant women^37^. To date there are no published reports on adipose expression of Cd36 in women with GDM, however it has been reported that placental expression of Cd36 is reduced in GDM compared to normal pregnancies^38^. Taken together the mRNA expression data indicates that normal pregnancy alters adipose tissue metabolism and that GDM like conditions, as observed in P-HFHS dams diminish these changes leading to altered pregnancy associated lipid metabolism compensation. It should also be noted that our data indicate these changes are specific to pregnancy and not diet alone.

Women with GDM are known to have increased lipolysis later in pregnancy^3–6^. Here we show that P-HFHS dams late in pregnancy also have increased lipolysis. Previously we showed that P-HFHS have increased triglycerides as well as leptin levels^14^. Together with our previous reports^14^ the results presented here clearly indicate that exposure to brief HFHS diet just before and during pregnancy is able to induce adipose tissue dysfunction and altered lipid metabolism as observed in women with GDM.

Previously we have shown that ADM plays a role in mediating adipose tissue dysfunction in GDM^15,16^. Here we showed that ADM also may play a similar role in the altered adipose tissue dysfunction and lipid metabolism observed in P-HFHS dams. In women with GDM, expression of ADM and it’s receptor components, CRLR, RAMP2 and RAMP3 is elevated in term omental adipose tissue^15,16^. We showed that this increase in ADM signaling expression is regulated by glucose and tumor necrosis alpha^15^. ADM is also increased in the serum of GDM women^39^. Our previous studies also showed that ADM dose dependently stimulates lipolysis in human adipocytes and that ADM increased the expression of leptin and resistin in adipose tissue from normal pregnant women^15,16^. We also showed that increases in leptin and resistin observed in adipose tissue from GDM can be blocked by ADM antagonists^15^. Furthermore, ADM inhibited phosphorylation of insulin receptor β^15^. The data presented here also indicates that brief HFHS diet feeding 1 week before and during pregnancy results in increased Adm signaling in adipose tissue. Moreover, Adm was able to induce lipolysis in a dose dependent manner in VAT from pregnant mice further supporting a role for Adm in lipid metabolism during pregnancy. However, the more detailed signaling mechanisms of Adm induced lipolysis require additional studies, and our animal model can be a useful tool in understanding the mechanistic role Adm may play in the altered lipid metabolism associated with GDM.

Taken together we demonstrated that exposure to brief HFHS diet 1 week before and during pregnancy results in altered energy balance and lipid metabolism as well as adipose tissue dysfunction. We further demonstrated that increased Adm signaling may play a role in this altered adipose dysfunction and that this animal model maybe useful in further understanding the role of Adm in the pathophysiology of GDM. Further work is needed to elucidate the mechanisms through which Adm is regulating increased lipolysis in GDM. The data presented here further strengthens our mouse model as useful tool for GDM research. Future work will also focus on screening Adm antagonists as well as other compounds that can be useful to develop more effective treatment options for GDM.

## Data Availability

All data is provided in the manuscript for scientific evaluation.
